# Factors related to excessive out-of-pocket expenditures among the ultra-poor after discontinuity of PBF: a cross-sectional study in Burkina Faso

**DOI:** 10.1186/s13561-020-00293-w

**Published:** 2020-11-14

**Authors:** Yvonne Beaugé, Valéry Ridde, Emmanuel Bonnet, Sidibé Souleymane, Naasegnibe Kuunibe, Manuela De Allegri

**Affiliations:** 1 Heidelberg Institute of Global Health, Medical Faculty and University Hospital, Heidelberg University, Im Neuenheimer Feld 365, 69120 Heidelberg, Germany; 2 IRD (French Institute For Research on sustainable Development), CEPED (IRD-Université de Paris), ERL INSERM SAGESUD, Paris, France; 3 French Institute for Research on Sustainable Development (IRD), Unité Mixte Internationale (UMI) Résiliences, Paris, France; 4 UFR SDS EDS Université Ouaga 1 Professor JKZ, IRD (French Institute for Research on sustainable Development), AGIR - Global Alliance for Resilience, Paris, France; 5 Department of Economics and Entrepreneurship Development Studies, Faculty of Integrated Development Studies, University for Development Studies, Wa, Upper West Region, Ghana

**Keywords:** Out-of-pocket expenditure, Ultra-poor, Targeting, Performance-based financing, Burkina Faso

## Abstract

**Background:**

Measuring progress towards financial risk protection for the poorest is essential within the framework of Universal Health Coverage. The study assessed the level of out-of-pocket expenditure and factors associated with excessive out-of-pocket expenditure among the ultra-poor who had been targeted and exempted within the context of the performance-based financing intervention in Burkina Faso. Ultra-poor were selected based on a community-based approach and provided with an exemption card allowing them to access healthcare services free of charge.

**Methods:**

We performed a descriptive analysis of the level of out-of-pocket expenditure on formal healthcare services using data from a cross-sectional study conducted in Diébougou district. Multivariate logistic regression was performed to investigate the factors related to excessive out-of-pocket expenditure among the ultra-poor. The analysis was restricted to individuals who reported formal health service utilisation for an illness-episode within the last six months. Excessive spending was defined as having expenditure greater than or equal to two times the median out-of-pocket expenditure.

**Results:**

Exemption card ownership was reported by 83.64% of the respondents. With an average of FCFA 23051.62 (USD 39.18), the ultra-poor had to supplement a significant amount of out-of-pocket expenditure to receive formal healthcare services at public health facilities which were supposed to be free. The probability of incurring excessive out-of-pocket expenditure was negatively associated with being female (β = − 2.072, *p* = 0.00, ME = − 0.324; *p* = 0.000) and having an exemption card (β = − 1.787, *p* = 0.025; ME = − 0.279, *p* = 0.014).

**Conclusions:**

User fee exemptions are associated with reduced out-of-pocket expenditure for the ultra-poor. Our results demonstrate the importance of free care and better implementation of existing exemption policies. The ultra-poor’s elevated risk due to multi-morbidities and severity of illness need to be considered when allocating resources to better address existing inequalities and improve financial risk protection.

**Supplementary Information:**

The online version contains supplementary material available at 10.1186/s13561-020-00293-w.

## Introduction

In Burkina Faso, the provision of most adult curative services is still subject to the payment of user fees at the point of use. User fees can be imposed on drugs, medical material, entrance fees or consultation fees, exposing many but especially the ultra-poor to a high risk of catastrophic expenditure. The ultra-poor or so-called ‘indigents’ are a recognized category of the population in Burkina Faso, representing the most disadvantaged part of the society [1, 2]. The Ministry of Social Action and National Solidarity defines indigents as people who are without any social or economic means on a sustained basis and unable to care for themselves [3]. Accordingly, any user charges whether high or low are likely to exceed the ultra-poor’s financial means [4–6] forcing them to sell the few assets they might possess, borrow money or decide to forego the required healthcare [7, 8].

The government of Burkina Faso has recognized the need for exemption policies to better protect the most vulnerable against the economic impact of illness. Many policies have been adopted over the years to guarantee free healthcare to the ultra-poor on the full range of essential services [9, 10] including the new compulsory universal health insurance scheme (RAMU) with its legislation adopted in September 2015. However, the actual implementation of these measures for the ultra-poor has mostly lagged behind political commitment [9–11]. Alongside the formulation of new policies, the government of Burkina Faso, together with its development partners, has launched several exemption pilots to protect the poor from the financial burden induced by user fees [12–14].

In 2014, a performance-based financing intervention (PBF) in combination with user fee exemptions for the ultra-poor was implemented in eight districts [14, 15]. Community-based targeting (CBT) was used to identify up to 20% of the poorest population living in the health facility catchment area. Community committees selected the ultra-poor in their villages [16]. Upon completion of the targeting process (January 2016), every identified ultra-poor was meant to receive an exemption card, allowing them to receive free basic healthcare services at all public healthcare facilities [17]. The user fee exemptions did not cover transport cost to the facility. The intervention officially ended in June 2018. A transition to the next World Bank project had been planned, but not yet realized at the time of writing this manuscript [18].

To our knowledge, there has not been any study measuring out-of-pocket expenditure (OOPE) among the targeted ultra-poor to track progress towards financial risk protection in Burkina Faso. Only a few studies are available that looked into the level and determinants of OOPE in Burkina Faso for the general population. This was reported to range between FCFA 8404 (USD 17.4) [19] and FCFA 9362.52 (USD 15.7) [20]. Su et al. (2006) reported that as much as 14.6–25.7% of the households from the lowest quartile in the general population in Nouna incurred catastrophic healthcare expenditure before the implementation of community-based health insurance [21]. None of these studies measured the extent to which ultra-poor are exposed to financial hardship through the use of health services, despite researchers have highlighted the importance of monitoring such outcome to secure the achievement of the sustainable development goals (SDGs), in particular SDG3, targeting specifically health for all [22]. The lack of evidence on the financial risk protection for the ultra-poor in Burkina Faso is comparable with other low-and middle income countries. Only Jacobs et al. (2007) found that in Cambodia, fee exempted patients paid on average USD 4.3 per healthcare visit which is USD 9 less than fee-paying patients [23]. Looking at evidence from Zambia, Masiye et al. (2016) and Lepine et al. (2017) reported an important reduction in medical expenses for the general population after the introduction of the nationwide user fee removal [24–26]. However, both studies highlighted that the effect of user fee exemptions might not reach the poorest proportionately.

Our study aimed at filling this knowledge gap by using cross-sectional data to assess the magnitude of OOPE on formal healthcare services among the ultra-poor who had been targeted and exempted within the context of the performance-based financing intervention in Burkina Faso. Moreover, we aimed at estimating the factors that explain the ultra-poor’s probability of incurring excessive OOPE. We defined ‘excessive spending’ as having expenditure greater than or equal to two times the median OOPE [27]. In 2019, the year in which we conducted our study, the ultra-poor in the study region were only equipped with exemption cards under the PBF program and not RAMU (RAMU was not yet operational in the study district). Our study findings are intended to inform policy makers on the protective effect of targeted user fee exemptions from the cost of illness for the ultra-poor.

## Methods

### Study setting

The study was conducted in Diébougou District, in Bougouriba Province in the South-West region of Burkina Faso, one of the eight districts where PBF was combined with targeting and exemption of the ultra-poor. In 2017, the district had a total population of 139,824, with over 40% living below the national poverty line [28]. Diébougou has 24 functioning government healthcare facilities (4 dispensaries, 19 Primary Healthcare facilities (CSPS) and one district hospital) with a total of eight general practitioners and two pharmacists [29]. In 2016, the average annual number of healthcare contacts per inhabitant was 1.68 [29], which is high compared to the country-wide average of 1.02 contacts. The CBT process identified 6034 people in Diébougou as being ultra-poor in 2015, which related at that time to about 9% of the district’s population [30]. In early February 2016, the district management received the exemption cards for further distribution via the CSPS to the ultra-poor.

### Data and their sources

The study used a cross-sectional dataset of 292 ultra-poor individuals living in the Diébougou health district, previously identified by a study conducted in 2015 [31]. Originally, a three-stage random procedure was applied to identify study individuals across different PBF districts with targeting in Burkina Faso, described in detail elsewhere [31]. In brief, at the first stage, four out of eight districts were selected; at stage two, villages with more than ten ultra-poor people were selected; at stage three, only ultra-poor aged 18 and above and whose name was on the original ultra-poor list were recruited for the survey [31]. In 2019, the survey could only be conducted for the selected ultra-poor in Diébougou district, with the specific aim of understanding what happened to the selected ultra-poor post-PBF and prior to all new policies being launched. The survey was administered from June 10th to June 25th, 2019 by five trained enumerators fluent in the local language under the supervision of a study coordinator. Data were collected digitally using tablets. The survey included the following five sections: identification of the indigent including geo-location, socio-demographic information, exemption card and health service utilisation, illness-reporting and healthcare needs, functional capacities and support network.

### Variables and their measurement

Table 1 reports all variables, their measurement, and the hypothesized sign of the association with the outcome variable. Table 2 lists OOPE on formal healthcare services, transportation to receive formal healthcare services and total OOPE.

**Table 1:** Variables, their measurement and hypothesized direction of the coefficient

Outcome Variables	Measurement	**Hypothesized** direction of the coefficient
Excessive OOPE on formal healthcare services	Dichotomous 1 if excessive 0 otherwise	
**Explanatory Variables**		
**Binary**		
Sex	0 = Male	+
	1 = Female	
Educational level	0 = No education	–
	1 = Education	
Exemption card	0 = No	–
	1 = Yes	
Marital status	0 = All else	–
	1 = Married	
Relation to the household head	0 = All else	+
	1 = Household head	
Perceived health	0 = All else	–
	1 = Good	
Disability	0 = No	+
	1 = Yes	
**Continuous**		
Age	Years	+
Household size	Household member	+
Distance to the nearest healthcare center (in km)	Km	+
**Categorical**		
Poverty index	1 Poorest	+
	2 Medium Poor	+−
	3 Least poor	–

**Table 2:** Socio-demographic characteristics of the study population

	Sample N = 110 individuals (100%)	
	N	%
Excessive OOPE on healthcare services when utilising formal health care services		
No	11	7.27
Yes	99	92.73
Exemption card		
No	18	16.36
Yes	92	83.64
Sex		
Male	43	39.09
Female	67	60.91
Educational level		
No education	96	87.27
Education	14	12.73
Marital Status		
All else	53	48.18
Married	57	51.82
Relation to the household head		
All else	75	68.18
Head of household	35	31.82
Perceived Health		
All else	89	80.91
Good	21	19.09
Disability		
No	80	72.73
Yes	30	27.27
Poverty Index		
Poorest	32	29.09
Medium poor	38	34.55
Least poor	40	36.36
	**Mean**	**Sd**
Age (in years)	55.11	18.67
Household size	14.25	11.54
Distance to nearest healthcare centre (in km)	4.45	4.75

Our primary outcome variable was excessive OOPE for formal healthcare services without transportation costs. Transportation cost were excluded because the user fee exemptions did not cover transportation cost. Formal healthcare services refer to curative healthcare services sought by the respondent either at the primary healthcare centres (CSPS) or district-level hospitals. As we did not have information about household consumption or income (study population = ultra-poor without financial means), it was not possible to measure catastrophic expenditures. The direct OOPE for formal healthcare of the ultra-poor was dichotomised (0 = no excessive spending, 1 = excessive spending). The category 0 = no excessive spending includes the zeros, i.e. the ones treated for free due to exemption cards, while the category excessive spending, captures OOPE above a given threshold. Our main explanatory variable was the exemption card. It is a dichotomised variable and refers to whether a respondent has received a user exemption card that he/she could present at the health centre to receive free care within the context of the PBF intervention.

In this study, we used several covariates to control for demographic and socio-economic characteristics and health status. We decided to dichotomize most of our variables due to the relatively small sample size, which made it possible to keep an adequate sample in each of our categories. The dichotomization also helped to focus the statistical analysis around the two comparison groups we expected to differ. Demographic characteristics included sex, age, marital status, relation to the household head, and household size and socio-economic factors included educational level. Sex was a dichotomous variable (male/female). Age (in years) was a continuous variable. Marital status was a categorical variable and contained five categories (single, monogamous married, married polygamous, widowed, divorced/separated). The original variable was dichotomised (All else and married). We did so to show the vulnerability associated with being unmarried. Status in the household was a categorical variable. The original variable composed of 11 categories (Household head; spouse; brother/sister; son/daughter; nephew/niece; Grandson/daughter; father/mother; cousin; son/daughter in law; mother/father-in-law; other parent; other link). The variable was dichotomised to show the superiority of household heads in the use of resources. Educational level was a categorical variable with 16 categories (1 none; 2 nursery school; 3 CP1 4 CP2; 5 CE1 6 CE2; 7 CM1; 8 CM2; 9 Sixième; 10 Cinquième; 11 Quatrième; 12 Troisième 13 Seconde; 14 Première; 15 Terminale; 16 Supérieur). We dichotomised the variable (no education and education) as done by previous studies [32, 33]. We did so because the educational level of the ultra-poor people is generally very low. Only 12.73% of our study sample (ultra-poor population) reported any form of education. Respondents with higher education than nursery school were assigned to the category 1 ′Education′.

As a proxy for health status, we used self-rated perceived health and disability to control for the participant’s health condition, which can affect healthcare spending. Self-rated perceived health was a categorical variable (good, medium, bad) and was dichotomised (All else/Good). Disability was a dichotomous variable (Yes/No). The variable distance was continuous. We also computed a poverty index using Principal Component Analysis (PCA) on durable asset ownership and housing characteristics specific for this rural location. This approach allowed classifying the ultra-poor from the poorest (1) to the least poor (3), to capture socio-economic differences among them.

The hypothesized direction of the co-efficient was informed by previous evidence on factors associated with high and catastrophic OOPE among poor and vulnerable groups [20, 21, 24, 34–37]. In particular, we expected women, the uneducated, unmarried and respondents in the lowest quintile to be more vulnerable towards an increased risk of excessive spending [21, 36]. An older age, bad health status and a disability was also expected to contribute to an elevated risk to excessive spending, since an increased age and a bad health condition contribute to a higher need of healthcare [20, 35]. Likewise, we expected a greater household size to contribute to an elevated risk of excessive spending since they might experience more illness [21]. At the same time large households are more likely to have elderly people in their union who carry an elevated risk for healthcare. Exemption card ownership was expected to lower the probability of excessive spending [27]. Household heads were expected to be more likely to spend excessively since prior work has indicated that they are more likely to seek care and to incur higher OOPE than other household members, as their health is essential for household’s survival [38–40]. Living remotely from the health facility was expected to be positively associated with excessive spending since ultra-poor rather seek formal healthcare at remote health facilities when illness is already very severe requiring complex treatment [24, 37].

### Analytical approach

For our analysis, we used the truncated sample of respondents who utilised formal healthcare services at the healthcare facility conditional upon illness reporting in the prior six months (*N* = 110). We did so because the user fee exemption cards were earmarked only to healthcare services provided by formal healthcare facilities. First, we applied descriptive statistics to identify sample distribution for all variables included in the analysis. We calculated the mean, standard deviation (SD), median, range values for OOPE on formal healthcare services, transportation to receive formal healthcare services and overall OOPE. All expenditure variables were recorded in FCFA (FCFA 1 = 0.0017 USD). Extreme values of the dataset were first graphically investigated by using boxplots. We did not screen out and included in our datasets three illness episodes which had resulted in OOPE above FCFA 100,000 (USD 173). We cross-checked the nature of these extreme values with study coordinator and enumerators who confirmed their validity. These extremely high OOPE refer to ultra-poor who had been evacuated for surgeries. The costs were covered by family and in particular by adult children living abroad (Ivory Coast and Ghana). Although these poor people received external support and might not comply with the previously mentioned definition of ultra-poor, they were initially identified as such and therefore not excluded from our regression analysis as they are accurate representations of community reality. We displayed mean OOPE using four different scenarios: OOPE, including zeros and outliers, excluding only zeros, excluding only extreme values and excluding zeros and extreme values.

For the regression analysis, however, we used the dichotomised variable ‘Excessive OOPE on formal healthcare services’. We used three different thresholds: 1. “high expenditure”, 2. “medium-high expenditure” and 3. “extremely high expenditure”. We used threshold 1 for the main model and threshold 2 and 3 for sensitivity analysis. As done by authors in previous studies [41], we defined “High expenditure” as having expenditure greater than or equal to two times the median; “Medium-high expenditure” as having expenditure greater than or equal to the median; and “Extremely high expenditure,” as having expenditure greater than or equal to three times the median.

Given that we classified the outcome of interest as binary, $$ {y}_i=\left\{\ \begin{array}{c}1\  if\ {y}^{\ast }>0\\ {}0\  otherwise\end{array}\right. $$; where *y*^∗^ = *x*_*i*_*β* + *μ*_*i*_ (1), multivariate logistic regression was performed to investigate the factors related to excessive OOPE among the ultra-poor on formal healthcare services for a single illness-episode within the last six months. From equation (1), *y*^∗^ is the observed excessive health-care expenditure, *x*_*i*_ represents individual respondent characteristics as presented in Table 1, *β* is the coefficients of *x*_*i*_ while *μ*_*i*_ is a symmetrically distributed error term. Following the literature [42, 43], equation (1) was estimated using the maximum likelihood estimation procedure, and the marginal effects were calculated for each *x*_*i*_ to derive the magnitudes of effect of the individual characteristics on the probability of a respondent incurring excessive health-care expenditure, while holding all other covariates constant.

We further geolocated the respondents and transferred their GPS information into a Geographic Information System to better understand patterns between the residential location of the respondents and CSPS. We applied point analysis (location of ultra-poor) and the kernel density estimator method. The densities represent the concentration of selected ultra-poor within a radius of 2000 m.

## Results

### Socio-demographic characteristics of the study population

Table 2 provides descriptive statistics, frequencies and percentages for the study sample.

**Fig. 1:**
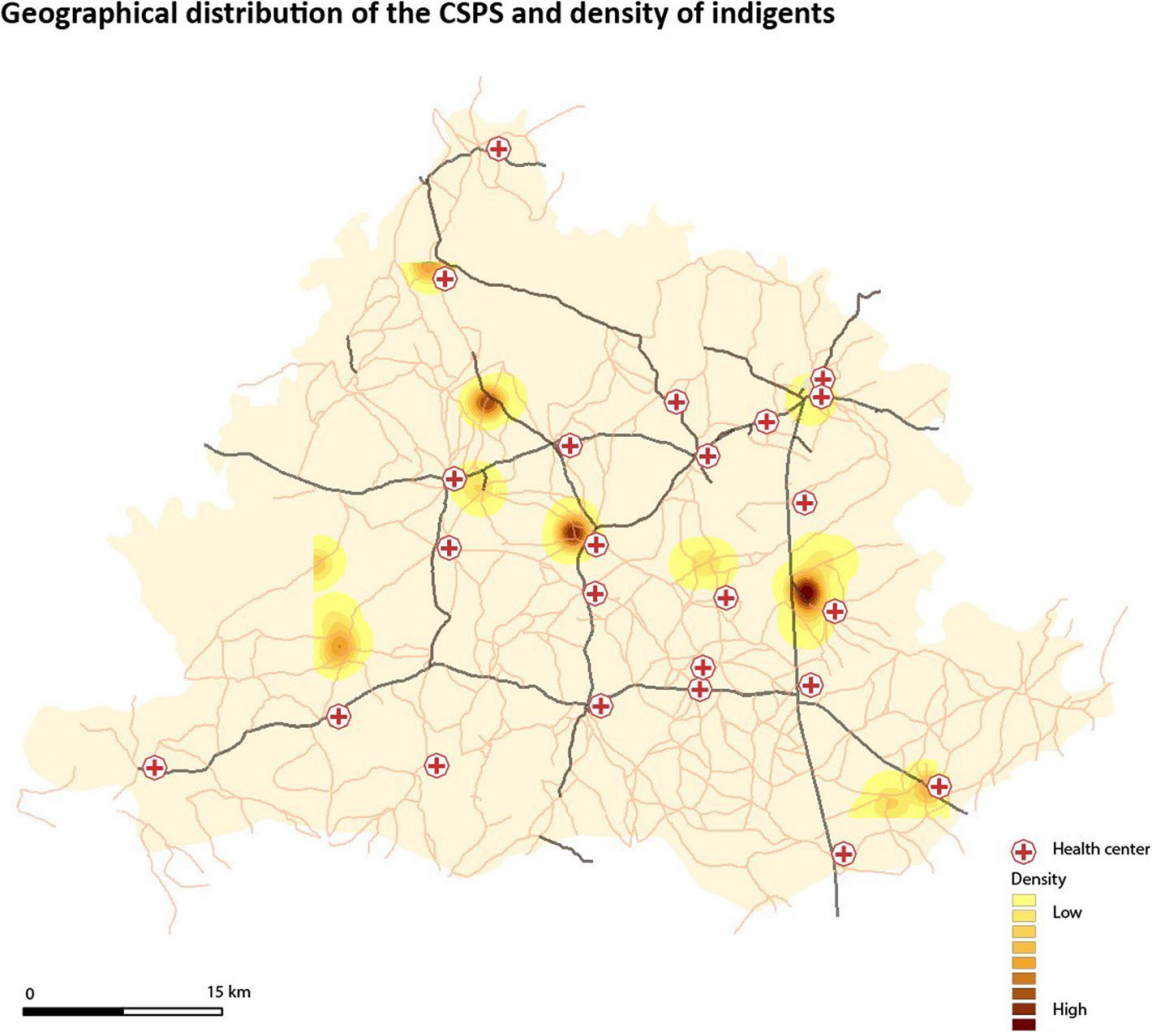
Geographical distribution of the CSPS and ultra-poor

The majority of the sample, 60.91% were females with a mean age of 55.11 years. Only 12.73% attained formal education. Half of the sample was married. About one-third of the study sample was the household head. Being in good health was reported by only 19.09% and being disabled by 27.27%. Respondents lived in rather big households with an average of 14 household members which is typical for rural Burkina Faso. Over 80% reported having received an exemption card. 29.09% belonged to the poorest, 34.55% to the medium poor poverty quantile and 36.36% to the poorest quintile. The mean distance from the respondent’s home to the nearest healthcare facility was 4.45 km. Figure 1 illustrates the mixed picture of the geographical concentration, whereby some of the respondents are concentrated around the primary healthcare facilities but also in remote areas.

### OOPE on formal healthcare services and transportation

Table 3 illustrates the mean OOPE for formal healthcare, transportation and total for respondents who reported an illness episode within the last six months. The information is shown for four possible scenarios:

**Table 3:** OOPE for formal healthcare services and transportation in FCFA

OOPE	N	%	Mean	SD	Median	Min	Max
1. **Scenario: Including zeros and outliers**							
Formal healthcare	110	100.00	20,424.45	81,552.69	5000	0	700,000
Transport	49	26.77	2134.18	2377.49	1400	0	12,000
TOTAL	110	100.00	21,375.14	82,647.95	5050	0	710,000
2. **Scenario: Excluding only outliers** ^**a**^							
Formal healthcare	107	97.27	8847.57	10,838.98	5000	0	60,000
Transport	48	43.64	1928.65	1912.758	1400	0	10,000
TOTAL	110	100.00	9447.86	11,196.48	5000	0	62,000
3. **Scenario: Excluding only zeros** ^**b**^							
Formal healthcare	99	90%	22,693.84	85,704.95	5100	500	700,000
Transport	48	43.64	2178.646	2381.975	1450	500	12,000
TOTAL	102	92.73	23,051.62	85,631.19	5850	500	710,000
4. **Scenario: Excluding zeros and outliers** ^**c**^							
Formal healthcare	96	87.27	9861.35	10,999.28	5000	500	60,000
Transport	47	42.73	1969.68	1911.96	1400	500	10,000
TOTAL	102	92.73	10,188.87	11,298.93	5600	500	62,000

Note: Of the 110 respondents using formal healthcare services, 11 reported zero expenditures

^a^ excluding three observations through trimming top 3% cutoff =60,000 for formal healthcare; excluding one observation through trimming top 3% cutoff = 10,000 for transport

^b^ excluding 11 observations with zeros for formal healthcare and excluding one observation with zeros for transport

^c^ excluding 11 observations with zeros for formal healthcare; excluding one observation with zeros for transport: excluding three observations through trimming top 3% cutoff =60,000 for formal healthcare; excluding one observation through trimming top 3% cutoff = 10,000 for transport

The mean OOPE for formal healthcare services when including zeros (exempted ultra-poor were supposed to be treated for free) for *N* = 110 was FCFA 20424.45 (USD 34.72) while FCFA 2134.18 (USD 3.62) was spent on transportation for *N* = 49. In comparison, when excluding zeros and extreme cases, the OOPE on formal healthcare services for *N* = 96 was FCFA 9861.35 (USD 16.76), while FCFA 1969.68 (USD 3.35) was spent on transportation for *n* = 47. For scenario 1, the total amount was FCFA 21375.14 (USD 36.34) and FCFA 10188.87 (USD 17.32) for scenario 4. The median OOPE across the two scenarios amounted to about FCFA 5000–5850 (USD 8.50–9.95).

In Table 4 we calculated the prevalence of excessive expenditure among the ultra-poor and the average OOPE for the different thresholds. Using the high expenditure threshold, 29.09% of the respondents with an illness episode had excessive expenditures.

**Table 4:** Prevalence of excessive expenditure and mean OOPE for different thresholds

Excessive OOPE threshold	No. of respondents	% of respondents with illness N = 110	Mean high OOPE for formal healthcare services mean (SD) in FCFA
High expenditure	35	29.09	56,762.86 (138,984.3)
Medium high expenditure	58	52.73	36,684.91 (110,213.7)
Extremely high expenditure	27	24.55	70,316.67 (156,285.4)

### Results from the regression model on factors related to excessive OOPE

Table 5 presents the results of the logistic regression exploring the factors related to excessive OOPE at the individual level. We first present the results from our main model using ≥2 times the median OOPE as a cut-off point for high expenditure.

**Table 5:** Results from the regression model exploring the factors related to excessive OOPE at the individual level

Variable	Main model = Excessive OOPE on formal health care services N = 110					
	Regression coefficient	*p-*value	[95% CI]	Marginal effects	*p*-value	[95% CI]
Exemption card owner	−1.787	**0.025**	−3.350 -0.224	−0.279	**0.014**	−0.503 -0.057
Female	−2.072	**0.003**	−3.440 -0.705	−0.324	**0.000**	−0.501 -0.148
Educated	−1.703	0.158	−4.068 0.662	−0.267	0.145	−0.625 0.092
Married	0.192	0.738	−0.932 1.315	0.030	0.738	−0.146 0.206
Head of household	− 0.943	0.160	−2.256 0.371	−0.148	0.146	−0.346 0.051
Good health status	−1.913	0.084	−4.082 0.256	−0.299	0.074	−0.628 0.030
Having a disability	0.295	0.593	− 0.787 1.377	0.046	0.592	−0.122 0.215
Age	0.036	0.061	− 0.002 0.074	0.006	**0.047**	0.000 0.011
Household size	−0.030	0.211	−0.078 0.017	−0.005	0.199	−0.012 0.002
Distance	−0.080	0.195	−0.201 0.041	−0.012	0.184	−0.031 0.006
Poverty Index (vs. 1 = ultra-poor)						
2 = Medium poor	0.069	0.914	−1.175 1.313	0.010	0.914	−0.174 0.194
3 = Least poor	0.568	0.383	−0.709 1.844	0.089	0.371	−0.105 0.283
_cons	0.886	0.616	−2.577 4.348			
LR chi2(12)	33.71					
Prob > = chibar2	0.001					

We found that having an exemption card had a protective effect against excessive OOPE in this ultra-poor population. The probability of incurring excessive OOPE decreased by 28% for those who received an exemption card. We also found that the probability of excessive OOPE decreased by 32% if the respondent was a woman. All other factors included in the main model were insignificant. The results remained stable throughout the two models chosen for sensitivity analysis, where we used the medium and extreme high expenditure threshold. Interestingly, the factor age significantly increased the probability of incurring an excessive expenditure only in model 2 and 3 (see Additional files 1 and 2). In the main model, age was insignificant. The results also remained stable when excluding the three extreme cases (see Additional file 3).

## Discussion

Our study makes a unique contribution to the existing literature by investigating OOPE among the ultra-poor in Burkina Faso, a segment of society who lives in extreme poverty, is hardest to reach and thus often neglected within the scientific landscape as data is hardly available on these excluded individuals [44]. Accordingly, even a small dataset as ours is precious to closely track and understand the progress of these people and integrate the gained knowledge into the planning and prioritizing of future interventions to leave no one behind as envisioned in the 2030 Agenda for Sustainable Development. Our study is the first, which assesses the magnitude of OOPE on formal healthcare services among targeted and exempted ultra-poor people. In light of the surprisingly high expenditure of the ultra-poor, we also aimed at estimating the factors that explain the ultra-poor’s probability of incurring the excessive OOPE. The findings of our study offer valuable practical and political implications for countries currently moving towards a national health insurance scheme with the aspiration also to include the weakest members of the society. Yet, due to the small sample size, the result should be interpreted with caution.

The first crucial finding of our study was that 90% of our study population incurred expenditure above zero, while only 10% reported zero expenditure. Most striking is that these identified and former exempted “ultra-poor” had to pay a substantial total mean of FCFA 23051.62 (USD 39.19) towards expenses to cover their formal healthcare costs for a single illness-episode within the last six months. In contrast, Beogo et al. (2016) assessed the mean OOPE for public health services among individuals living in the capital of Burkina Faso at FCFA 8404 (USD 14.29) [19]. Nakovics et al. (2019) used household-level data for 24 districts (a third of the country) and calculated overall OOPE of FCFA 9362.52 (USD 15.92) (irrespective of the type of care used) for the general rural population [20]. The lowest socio-economic quintile in the study done by Nakovics reported OOPE at the same level as the rest of the population [20]. What is obvious is the discrepancy of our values with those of previous studies. Here it is essential to note that our calculation included three extreme, but validated cases where ultra-poor got evacuated for surgery with extremely high accompanying costs. When we removed these cases, the mean was calculated at FCFA 10188.87 (USD 17.32) almost matching the reported mean by Beogo et al. (2016) and Nakovics et al. (2019). Irrespective of the approach taken, both amounts USD 39.19 and USD 17.32 impose a dramatic economic burden on the ultra-poor people who already live below the national poverty line of USD 1.90 a day [45]. Additionally, these numbers are a demonstration of the current inequitable health financing mechanisms in Burkina Faso.

Our study also reveals that almost half of those who seek formal healthcare services (45%) incurred a positive expenditure on transport costs with an average of FCFA 2178.65 (USD 3.70). Not only do more ultra-poor incur transport costs, but at the same time, the average cost is 27% higher than what the general residents in rural Burkina Faso pay for transport for healthcare (FCFA 1670.83) (USD 2.84) [20]. This finding seems entirely plausible at first sight as it is known that ultra-poor usually live socially isolated in remote areas [46] and do not own private vehicles (e.g. bicycle, motorbike or donkeys) to get to the health center and that might lead to an increased need to use other means of transport that drives costs up. The map (Fig. 1) of the distribution of the CSPS and density of indigents also demonstrated the geographical remoteness. However, when comparing the mean difference of the general rural resident and the identified ultra-poor from their residential spot to the nearest health facilities, we do not see a big difference which makes us assume that the distance alone might not be the main driver of the transport costs. Instead, we assume that their old age, the seriousness of the illness and a possible late-stage of seeking care (not able to walk, stand, sit alone without assistance) might demand that ultra-poor be transported in a specific way, e.g. making it necessary to have accompanied transportation with a borrowed vehicle (involving fuel costs) [32, 47, 48].

Looking specifically at the results of the regression models, it was striking to see that the exemption card, which respondents received in early February 2016 in Diébougou within the PBF intervention (3 years before the data collection), decreased the probability of incurring excessive OOPE by 28 percentage points. This finding shows the potential of the exemption in achieving financial protection for the poorest, which is a key objective of Burkina’s first health financing strategy (2017–2030). It is remarkable, especially against the background that the intervention ended in June 2018 with the end of the World Bank funding, where healthcare providers received last program reimbursements in January 2018. Our data collection started almost exactly one year after the official end of the project. While further qualitative studies are needed to clarify the specific reasons for this positive development, initial field feedback pointed towards the core of goodness in healthcare workers and their uptake of program ownership in relation to the user fee exemptions after discontinuity of PBF. Indeed, it is assumed that some health workers continue to feel responsible for their community’s health and show compassion and kindness towards the ultra-poor. As a result, they might encourage support actions in conjunction with the management committees or an autonomous manner, to provide the minimum package of healthcare services to the ultra-poor. We also refer to the exemption policy implemented by the government in 2009 which demonstrated that only asking health worker at the primary level to exempt the ultra-poor was never successful. An enabling mechanism (exemption cards) in combination with good will is necessary to allow the exemptions to be turned into practice. Similar developments have been noted by Ridde & Girard (2004), who described that some health personnel, in their good graces, continued to ensure exemption for healthcare for identified ultra-poor [49]. This is in line with Seppey et al. (2017) who described that after discontinuity of PBF in Mali it is mainly the activities with a higher degree of autonomously driven motivation that are more sustainable [50]. In the case of user fee exemptions, healthcare workers might be driven to continue to provide services to the ultra-poor even in the absence of project funding because doing so corresponds with their beliefs and values of equity, charity, justice and solidarity.

Furthermore, a positive association between age and excessive spending for formal healthcare services has emerged from the findings. This pattern is unsurprising and coherent with the broad literature [38] since an increasing age is a predisposing factor leading to higher rates of (multi)-morbidity and disability [51, 52]. Therefore, older people make substantial use of formal health services [53], require special diagnostics and consequently incur higher expenses [47, 54]. Similarly, we expected males to be more likely to spend excessively on formal healthcare services. The reasons are three-fold: first, Burkina Faso has been implementing several user fee exemptions and removal mechanism and policies targeting women including the launch of the gratuité policy in April 2016 to cover the healthcare fees for preventive and curative care for pregnant and lactating women which makes excessive spending less likely [55]. Secondly, as males are usually the breadwinner and their health essential for households’ survival, they might use formal healthcare services more compared to ultra-poor women [38–40]. Atchessi et al. (2016) pinpointed the prevailing power inequalities in gender relationships in this particular setting in Burkina Faso where decision-making power is usually with the men [47] which generally put women into a subordinate social position affecting their access to scarce resources [39].

### Methodological considerations

Although this study provides novel findings on OOPE amongst the ultra-poor, we need to acknowledge certain limitations. First, we acknowledge the relatively small size of our sample, and this necessitates a careful interpretation of results. Yet, we deem our results as essential since ultra-poor are severely understudied. We recommend replicating the study on a larger sample, albeit logistically complex. Secondly, no study has been conducted so far on the accuracy of the selection and targeting process (teasing out false-positive cases) of this specific scheme. Hence, we had no means of deciding on inclusion or exclusion of single cases. However, we carried out several sensitivity analyses by excluding extreme cases and also using different thresholds for excessive expenditure. Results stayed robust throughout. Thirdly, our study used self-reported information on illness reporting and expenditure data that could have been subject to recall bias, hence we cannot assure 100% accuracy of this data. Due to restriction by the dataset, we were not able to disaggregate OOPE from other cost items other than general spending on formal healthcare services and transportation. Despite these limitations, this paper provides essential evidence on the economic burden of out-of-pocket expenditure on the ultra-poor.

## Conclusion

Robust monitoring of OOPE among poor households is vital to understand improvements in financial protection and UHC [56]*.* To our knowledge, this is the first study examining the level of financial hardship among a targeted ultra-poor population in Africa. The evidence reviewed here highlights the high amount of OOPE that ultra-poor have to spend to cover their healthcare costs. We demonstrated that OOPE among the ultra-poor is at about the same level of people from higher socio-economic groups which is a clear demonstration of the unfairness of the current health financing schemes in Burkina Faso. When including valid extreme values, the ultra-poor on average even have higher expenditure than the general population most likely due to their old age, the severity of illness and complex medical profiles. The present study emphasizes that exemption cards had a protective effect against excessive OOPE despite the end of the intervention, which shows the relevance of free care for a vulnerable population. Policymakers must recognize the special needs of the ultra-poor for better tailored financial protection. A specific examination of service patterns of the ultra-poor is needed; the provision of enhanced and broadened coverage considering the elevated risks due to multimorbidity and chronic diseases of this sub-population is a logical consequence. Without considering these realities when allocating budgets, there is little prospect of making healthcare truly inclusive for the people living on the margin of society.

## Supplementary Information

**Additional file 1.** Sensitivity analysis: Results from the regression model exploring the factors related to excessive OOPE at the individual level using Medium-high expenditure threshold

**Additional file 2.** Sensitivity analysis: Results from the regression model exploring the factors related to excessive OOPE at the individual level using Extreme high expenditure threshold

**Additional file 3.** Sensitivity analysis: Results from the regression model exploring the factors related to excessive OOPE at the individual level, excluding the three extreme cases where ultra-poor had to accommodate over 100.000 FCFA to cover their healthcare costs.

## Data Availability

Due to the possibility of identifying respondents, the dataset cannot be made available open access. The authors are willing to share the database upon specific requests.

## Abbreviations

CBT: Community-based targeting
FCFA: Franc of the Communauté Financière Africaine
ME: Marginal effects
OOPE: Out-of-pocket expenditure(s)
PBF: Performance-based financing
CSPS (Centre de santé et de promotion sociale (Primary Healthcare Centers): Primary Healthcare facilities
PCA: Principal Component Analysis
RAMU: Régime d’assurance maladie universelle (universal health insurance scheme)
SDGs: Sustainable Development Goals
USD: United States dollars
UHC: Universal Health Coverage
