# Supplementary material for: Factors related to excessive out-of-pocket expenditures among the ultra-poor after discontinuity of PBF: a cross-sectional study in Burkina Faso
Source: Health Econ Rev. 2020 Nov 14;10:36. doi: 10.1186/s13561-020-00293-w (PMC7666767; doi:10.1186/s13561-020-00293-w)
Supplement: Supplementary file 1 — Additional file 1. Sensitivity analysis: Results from the regression model exploring the factors related to excessive OOPE at the individual level using Medium-high expenditure threshold [file 13561_2020_293_MOESM1_ESM.docx]

**Additional file 1**

Sensitivity analysis: Results from the regression model exploring the factors related to excessive OOPE at the individual level using Medium-high expenditure threshold

| **Variable** | **2nd model = Excessive OOPE on formal healthcare services**  N=110 | | | | | |
| --- | --- | --- | --- | --- | --- | --- |
|  | Regression coefficient | p-value | [95% CI] | Marginal effects | p-value | [95% CI] |
| Exemption card owner | -1.305 | **0.051** | -2.614 0.004 | -0.267 | **0.037** | -0.518 -0.017 |
| Female | -1.325 | **0.024** | -2.472 -0.178 | -0.271 | **0.013** | -0.486 -0.057 |
| Educated | -0.282 | 0.740 | -1.942 1.379 | -0.058 | 0.739 | -0.397 0.282 |
| Married | 0.464 | 0.324 | -0.459 1.388 | 0.095 | 0.316 | -0.091 0.281 |
| Head of household | -0.406 | 0.453 | -1.465 0.653 | -0.083 | 0.449 | -0.298 0.132 |
| Good health status | 0.893 | 0.139 | -0.289 2.076 | 0.183 | 0.125 | -0.051 0.417 |
| Having a disability | 0.081 | 0.869 | -0.884 1.047 | 0.017 | 0.869 | -0.181 0.214 |
| Age | 0.037 | **0.024** | 0.005 0.069 | 0.008 | **0.014** | 0.002 0.014 |
| Household size | 0.011 | 0.600 | -0.030 0.052 | 0.002 | 0.598 | -0.006 0.011 |
| Distance | 0.024 | 0.650 | -0.078 0.126 | 0.005 | 0.649 | -0.016 0.026 |
| Poverty Index  (vs. 1 = ultra-poor) |  |  |  |  |  |  |
| Medium poor | -0.227 | 0.673 | -1.282 0.828 | -0.047 | 0.673 | -0.264 0.170 |
| Least poor | 0.007 | 0.991 | -1.103 1.116 | 0.001 | 0.991 | -0.226 0.229 |
| _cons | -0.449 | 0.769 | -3.437 2.540 |  |  |  |
| LR chi2(12) | 21.22 |  |  |  |  |  |
| Prob >= chibar2 | 0.047 |  |  |  |  |  |
