# Supplementary material for: Factors related to excessive out-of-pocket expenditures among the ultra-poor after discontinuity of PBF: a cross-sectional study in Burkina Faso
Source: Health Econ Rev. 2020 Nov 14;10:36. doi: 10.1186/s13561-020-00293-w (PMC7666767; doi:10.1186/s13561-020-00293-w)
Supplement: Supplementary file 2 — Additional file 2. Sensitivity analysis: Results from the regression model exploring the factors related to excessive OOPE at the individual level using Extreme high expenditure threshold [file 13561_2020_293_MOESM2_ESM.docx]

**Additional file 2**

Sensitivity analysis: Results from the regression model exploring the factors related to excessive OOPE at the individual level using Extreme high expenditure threshold

| **Variable** | **3nd model = Excessive OOPE on formal healthcare services**  N=110 | | | | | |
| --- | --- | --- | --- | --- | --- | --- |
|  | Regression coefficient | p-value | [95% CI] | Marginal effects | p-value | [95% CI] |
| Exemption card owner | -1.635 | **0.046** | -3.240 -0.029 | -0.230 | **0.034** | -0.442 -0.017 |
| Female | -1.740 | **0.017** | -3.166 -0.313 | -0.244 | **0.008** | -0.425 -0.064 |
| Educated | -1.862 | 0.193 | -4.666 0.942 | -0.262 | 0.182 | -0.646 0.123 |
| Married | 0.365 | 0.560 | -0.861 1.591 | 0.051 | 0.558 | -0.120 0.223 |
| Head of household | -0.644 | 0.367 | -2.043 0.755 | -0.090 | 0.362 | -0.284 0.104 |
| Good health status | -1.415 | 0.211 | -3.633 0.802 | -0.199 | 0.204 | -0.506 0.108 |
| Having a disability | 0.615 | 0.284 | -0.511 1.741 | 0.086 | 0.276 | -0.069 0.242 |
| Age | 0.041 | **0.055** | -0.001 0.083 | 0.006 | **0.042** | 0.000 0.011 |
| Household size | -0.016 | 0.520 | -0.066 0.033 | -0.002 | 0.517 | -0.009 0.005 |
| Distance | -0.052 | 0.423 | -0.178 0.075 | -0.007 | 0.419 | -0.025 0.010 |
| Poverty Index  (vs. 1 = ultra-poor) |  |  |  |  |  |  |
| Medium poor | 0.009 | 0.989 | -1.308 1.327 | 0.001 | 0.989 | -0.172 0.174 |
| Least poor | 0.468 | 0.494 | -0.873 1.809 | 0.066 | 0.484 | -0.120 0.253 |
| _cons | -0.779 | 0.678 | -4.463 2.905 |  |  |  |
| LR chi2(12) | 27.57 |  |  |  |  |  |
| Prob >= chibar2 | 0.006 |  |  |  |  |  |
