# Supplementary material for: Factors related to excessive out-of-pocket expenditures among the ultra-poor after discontinuity of PBF: a cross-sectional study in Burkina Faso
Source: Health Econ Rev. 2020 Nov 14;10:36. doi: 10.1186/s13561-020-00293-w (PMC7666767; doi:10.1186/s13561-020-00293-w)
Supplement: Supplementary file 3 — Additional file 3. Sensitivity analysis: Results from the regression model exploring the factors related to excessive OOPE at the individual level, excluding the three extreme cases where ultra-poor had to accommodate over 100.000 FCFA to cover their healthcare costs. [file 13561_2020_293_MOESM3_ESM.docx]

**Additional file 3:**

Sensitivity analysis: Results from the regression model exploring the factors related to excessive OOPE at the individual level, excluding the three extreme cases where ultra-poor had to accommodate over 100.000 FCFA to cover their healthcare costs.

| Variable | Main model = Excessive OOPE on formal healthcare services  N=107 | | | | | |
| --- | --- | --- | --- | --- | --- | --- |
|  | Regression coefficient | p-value | [95% CI] | Marginal effects | p-value | [95% CI] |
| Exemption card owner | -1.969 | **0.017** | -3.586 -0.350 | -0.295 | **0.008** | -0.512 -0.078 |
| Female | -2.168 | **0.003** | -3.618 -0.717 | -0.325 | **0.000** | -0.505 -0.145 |
| Educated | -1.646 | 0.179 | -4.048 0.755 | -0.247 | 0.166 | -0.595 0.102 |
| Married | 0.085 | 0.888 | -1.094 1.264 | 0.013 | 0.888 | -0.164 0.189 |
| Head of household | -0.947 | 0.171 | -2.304 0.410 | -0.142 | 0.158 | -0.339 0.055 |
| Good health status | -1.779 | 0.112 | -3.972 0.414 | -0.267 | 0.103 | -0.587 0.053 |
| Having a disability | 0.206 | 0.721 | -0.924 1.336 | 0.031 | 0.720 | -0.138 0.200 |
| Age | 0.040 | **0.053** | -0.001 0.081 | 0.006 | **0.039** | 0.000 0.012 |
| Household size | -0.035 | 0.164 | -0.084 0.014 | -0.005 | 0.150 | -0.012 0.002 |
| Distance | -0.093 | 0.158 | -0.223 0.036 | -0.014 | 0.144 | -0.033 0.005 |
| Poverty Index  (vs. 1 = ultra-poor) |  |  |  |  |  |  |
| Medium poor | 0.211 | 0.753 | -1.100 1.521 | 0.0293 | 0.751 | -0.152 0.210 |
| Least poor | 0.746 | 0.272 | -0.585 2.078 | 0.1105964 | 0.252 | -0.079 0.300 |
| _cons | 0.822 | 0.656 | -2.789 4.432 |  |  |  |
| LR chi2(12) |  |  |  |  |  |  |
| Prob >= chibar2 |  |  |  |  |  |  |
